# Online chemical modeling environment (OCHEM): web platform for data storage, model development and publishing of chemical information

**DOI:** 10.1186/1758-2946-3-S1-P20

**Published:** 2011-04-19

**Authors:** I Sushko, AK Pandey, S Novotarskyi, R Körner, M Rupp, W Teetz, S Brandmaier, A Abdelaziz, VV Prokopenko, VY Tanchuk, R Todeschini, A Varnek, G Marcou, P Ertl, V Potemkin, M Grishina, J Gasteiger, II Baskin, VA Palyulin, EV Radchenko, WJ Welsh, V Kholodovych, D Chekmarev, A Cherkasov, J Aires-de-Sousa, Q-Y Zhang, A Bender, F Nigsch, L Patiny, A Williams, V Tkachenko, IV Tetko

**Affiliations:** 1 Institute of Bioinformatics and Systems Biology, Helmholtz Zentrum München - German Research Center for Environmental Health (GmbH), Ingolstädter Landstraße 1, D-85764 Neuherberg, Germany; 2 OCHEM consortium

## 

The Online Chemical Modeling Environment is a unique platform on the Web that aims to automate and simplify the typical steps required for QSAR modeling. The platform consists of two major subsystems: the database of experimental measurements and the modeling framework. The database is user-contributed and contains a set of tools for easy input, search and modification of thousands of records. The OCHEM database is based on the wiki principle and focuses on data quality and verification. The database is tightly integrated with the modeling framework, which supports all the steps required to create a predictive model: data search, calculation and selection of a vast variety of molecular descriptors, application of machine learning methods, validation, analysis of the model and assessment of the applicability domain. Our intention is to make OCHEM an ultimate platform to perform the QSPR/QSAR studies online and share it with other users on the Web. The OCHEM is free for the web users and it is available online at http://ochem.eu. “Computing chemistry on the web” [1] is becoming a reality.
